# Understanding the Steric Structures of Dicarboxylate Ions Incorporated in Octacalcium Phosphate Crystals

**DOI:** 10.3390/ma14112703

**Published:** 2021-05-21

**Authors:** Taishi Yokoi, Masakazu Kawashita

**Affiliations:** Institute of Biomaterials and Bioengineering, Tokyo Medical and Dental University, 2-3-10 Kanda-Surugadai, Chiyoda-ku, Tokyo 101-0062, Japan; kawashita.bcr@tmd.ac.jp

**Keywords:** octacalcium phosphate, dicarboxylate ions, layered structure, incorporation

## Abstract

Octacalcium phosphate (OCP) can incorporate various dicarboxylate ions in the interlayer spaces of its layered structure. Although not proven, these incorporated ions are believed to have a linear structure. In this study, the steric structures of twelve different dicarboxylate ions incorporated into OCP were investigated by comparing the experimentally determined interlayer distance of the OCP with the distance estimated using the molecular sizes of dicarboxylic acids calculated by considering their steric structures. The results revealed that the incorporated succinate, glutarate, adipate, pimelate, suberate, and aspartate ions possessed linear structures, whereas the incorporated azelate, sebacate, methylsuccinate, and malate ions exhibited bent structures. Further, the incorporated mercaptosuccinate ions featured linear, bent, other types of structures. Moreover, the steric structure of the incorporated malonate ion significantly differed from those of other dicarboxylate ions. The computational approach employed in this study is expected to deepen our understanding of the steric structures of dicarboxylate ions incorporated in the OCP interlayer spaces.

## 1. Introduction

Octacalcium phosphate (OCP, Ca_8_(HPO_4_)_2_(PO_4_)_4_·5H_2_O) is an inorganic compound with unique crystal–chemical characteristics, featuring a layered structure with apatitic and hydrated layers parallel to the (100) plane [1]. Monma and Goto incorporated succinate ions into the hydrated layers of OCP for the first time via the substitution of hydrogen phosphate ions (HPO_4_^2−^) [2]. Subsequently, various dicarboxylate ions were similarly incorporated into the OCP crystal structure [3,4,5,6,7,8,9,10] to enable precise control of the interlayer structure of OCP at the molecular level. OCP is the only compound in the family of calcium orthophosphates that exhibits this incorporation phenomenon, resulting in the formation of functional materials that are widely used as novel bone-repairing materials [11], aldehyde-specific adsorbents [12], and electrode materials for supercapacitors [13]. Consequently, the aforementioned phenomenon has garnered considerable attention from material scientists.

In OCP, the spacing between the (100) planes, (*d*_100_) increases upon the incorporation of dicarboxylate ions into the hydrated layers. Monma reported that the *d*_100_ linearly increases with the number of methylene groups (2 ≤ *n* ≤ 6) in the incorporated aliphatic dicarboxylate ions (^−^OOC(CH_2_)*_n_*COO^−^) [14]. Subsequent research on the molecular size of the incorporated dicarboxylate ions and the *d*_100_ of OCP was based on the following hypotheses. Firstly, the main chain of dicarboxylate ions incorporated in the hydrated layers of OCP is parallel to the OCP’s *a*-axis direction [14]. Secondly, the incorporated dicarboxylate ions have a linear structure [14]. As a result, OCPs with incorporated dicarboxylate ions having identical main chain structures were predicted to have identical *d*_100_. However, this prediction was disproven by the analyses of *d*_100_ in OCPs with incorporated succinic acid and its derivatives. Specifically, the OCPs with incorporated succinate, methylsuccinate, aspartate, malate, and mercaptosuccinate ions had *d*_100_ values of 21.4, 20.5, 21.3, 20.8, and 21.0 Å, respectively [14,15], despite these ions having identical main chain structures (*n* = 2). To determine the origin of this discrepancy, we re-examined the above hypotheses.

The first hypothesis was validated by the linear relationship between *d*_100_ and the length of the incorporated aliphatic dicarboxylate ions, determined by the number of methylene groups (2 ≤ *n* ≤ 6) [14]. Consequently, the second hypothesis based on the supposedly linear structures of the incorporated dicarboxylate ions was re-examined. Understanding the steric structures of the incorporated dicarboxylate ions will improve the molecular-level design of materials using OCP. Herein, we elucidate the relationship between the molecular sizes of dicarboxylic acids by considering their steric structures and the changes in the *d*_100_ of OCP. To our knowledge, this is the first study to analyse this relationship using a facile computational approach, presented in our previous work [16].

## 2. Calculation Method

**Figure 1:**
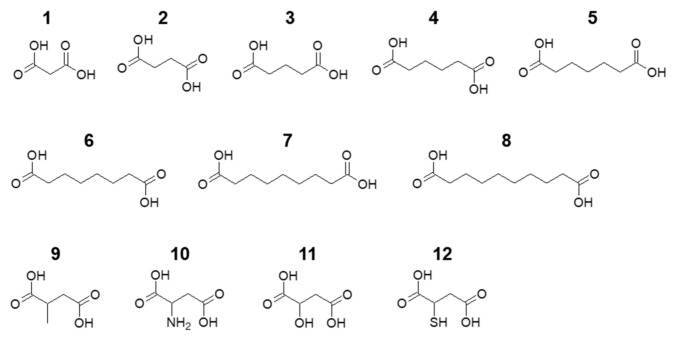
Molecular structures of the aliphatic dicarboxylic acids (1–8) and succinic acid derivatives (9–12) studied in this work: 1, malonic acid; 2, succinic acid; 3, glutaric acid; 4, adipic acid; 5, pimelic acid; 6, suberic acid; 7, azelaic acid; 8, sebacic acid; 9, methylsuccinic acid; 10, aspartic acid; 11, malic acid; 12, mercaptosuccinic acid.

The molecular sizes of the dicarboxylic acids were calculated by considering their steric structures using the computational approach described below. The molecular structures of the dicarboxylic acids being studied are shown in Figure 1. Their optimised ground-state structures in vacuum were determined using quantum chemical density functional theory (Firefly v. 8.2.0, Alex A. Granovsky) [17]. Notably, the structures of dicarboxylic acids in a vacuum rather than inside the OCP crystal were calculated, as the detailed structures of the dicarboxylate ions inside the OCP crystal have not yet been experimentally determined.

**Figure 2:**
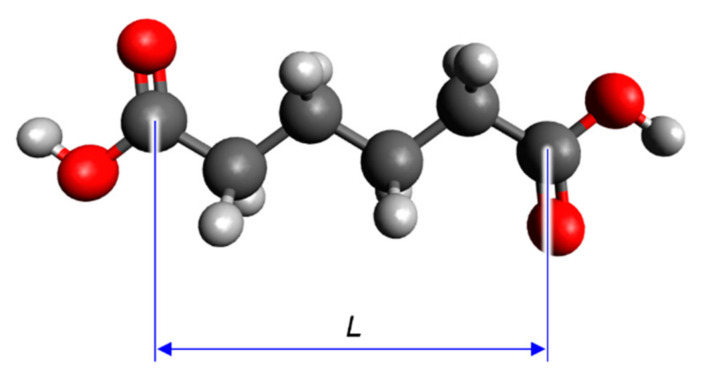
Schematic definition of *L* in the optimised structure of a carboxylic (in this case, adipic) acid.

The steric structures of the dicarboxylic acids were optimised using the Becke–three-parameter–Lee–Yang–Parr (B3LYP) hybrid functional [18,19,20,21] with the 6-31G(d) basis set. The correspondence of the steric structures with the local minima on the potential energy surfaces was confirmed by the calculation of the harmonic vibrational frequencies at the B3LYP/6-31G(d) level and the absence of imaginary frequencies. The distance between the carbon atoms of the carboxy groups in the optimised structures (*L*) was used as a parameter to express the molecular size of the dicarboxylic acids and was measured using an advanced molecular editor and visualiser (Avogadro v.1.2.0) (Figure 2) [22].

## 3. Results and Discussion

### 3.1. Validation for the Estimation of the Steric Structures of Incorporated Dicarboxylate Ions

**Figure 3:**
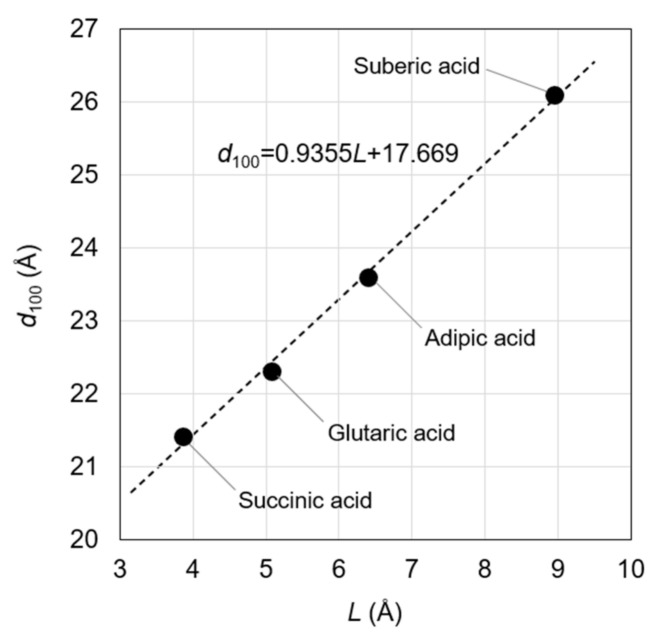
Relationship between *L* and *d*_100_ in OCP with incorporated carboxylate ions. Notably, the correlation coefficient (*R*^2^) of this approximate straight line is 0.9974.

The estimations were validated by reproducing the linear relationship between the *d*_100_ in OCP and the length of the incorporated aliphatic dicarboxylate ions (as defined in Figure 2). Such a linear relationship was previously observed in OCP with incorporated succinate, glutarate, adipate, pimelate, and suberate ions [14]. Based on the molecular size of pimelic acid, however, the *d*_100_ in the OCP with incorporated pimelate ions was lower than expected, which could be attributed to the insufficient amount of the incorporated pimelate ions. Hence, the current study focused on OCP with incorporated succinate, glutarate, adipate, and suberate ions, and correlated the *L* values of the related dicarboxylic acids with the *d*_100_ of the correspondingly incorporated OCP (Figure 3 and Equation (1)). For linear-chain succinic, glutaric, adipic, and suberic acids, the *L* values were calculated as 3.86, 5.07, 6.40, and 8.95 Å, respectively, with corresponding *d*_100_ values of 21.4, 22.3, 23.6, and 26.1 Å, respectively [14]. The linear relationship, manually derived by Monma from the steric structures of dicarboxylate ions, was consistent with the results of the quantum chemical calculations (Figure 3). Notably, the quantifiable nature of our method was superior to that of Monma [14]. The relationship between *L* and *d*_100_ was specifically expressed as:

*d*_100_ = 0.9355*L* + 17.669; *R*^2^ = 0.9974 (1)

The correlation coefficient (*R*^2^) of this relationship was found to be appreciable, indicating the successful fitting of the data. The *a*-axis length (19.87 Å) was different from the *d*_100_ (18.78 Å) of OCP [23], which was attributed to the host’s triclinic crystal structure. Furthermore, the conversion factor relating the *a*-axis length and the *d*_100_ (0.9451) was similar to the coefficient of *L* (0.9355) in Equation (1), which could be ascribed to the assumption that the dicarboxylate ions were parallel to the *a*-axis direction. Thus, the agreement between the two values validated our method for the estimation of the *d*_100_ from *L*. The agreement was confirmed in the range 21.3 Å ≤ *L* ≤ 26.0 Å, necessitating the careful use of Equation (1) in other ranges of *L*. Notably, the experimental value of *d*_100_ obtained using an X-ray diffractometer was successfully reproduced, even when the *L* values obtained from the structure of dicarboxylic acids optimized in a vacuum were used. Having established and verified the relationship between the *d*_100_ and *L* values, the steric structures of incorporated dicarboxylate ions are subsequently discussed using Equation (1).

### 3.2. Steric Structures of Incorporated Aliphatic Dicarboxylate Ions in OCP

The *d*_100_ values of the OCP containing aliphatic dicarboxylate ions were calculated from their *L* values using Equation (1) (*d*_100_(cal.)) and compared to those obtained experimentally (*d*_100_(exp.)) in a previous study (Table 1) [24]. As *d*_100_ is the sum of the thickness of the apatitic and the hydrated layers, the difference between the experimental and calculated *d*_100_ values (∆*d*_100_ = *d*_100_(exp.) − *d*_100_(cal.)) denotes a difference in the experimental and calculated thickness of the hydrated layer, because the thickness of apatitic layer is independent of dicarboxylate ion incorporation. Given that the main chain of the dicarboxylate ions incorporated in the hydrated layers of OCP is parallel to the *a*-axis direction and the thickness of the hydrated layer is determined by the size of dicarboxylate ions (namely *L* value) incorporated in the OCP interlayer, Δ*d*_100_ can be considered proportional to the difference in *L* values between the experimental and calculated dicarboxylic acid. Hence, ∆*d*_100_ was divided by *L* to obtain a useful index (∆*d*_100_/*L*). A low absolute value of ∆*d*_100_/*L* indicates similar *d*_100_(exp.) and *d*_100_(cal.) values. A positive ∆*d*_100_/*L* value indicates that *d*_100_(exp.) exceeds *d*_100_(cal.) and the structure of the incorporated dicarboxylate ions is stretched relative to the optimised structure. A negative ∆*d*_100_/*L* value indicates that *d*_100_(exp.) is lower than *d*_100_(cal.) and the structure of the incorporated dicarboxylate ions is compressed relative to the optimised structure. However, it is unlikely that the dicarboxylate ions were highly stretched or compressed in the OCP interlayers. If the calculation assumptions are correct, the absolute value of ∆*d*_100_/*L* should be within several percentage points. As the ∆*d*_100_/*L* values of succinate, glutarate, adipate, and suberate ions ranged from −2.3 to 3.0%, it was reasonable to assume that these ions adopted a linear conformation upon incorporation. For the pimelate ion, the ∆*d*_100_/*L* value was −5.4%, which suggests its absolute value to be slightly higher than the other dicarboxylate ions. The incorporation quantity (extent of HPO_4_^2−^ substitution) of pimelate ions was reported to be lower than that of succinate, glutarate, adipate, and suberate ions (Table S1) [24], which explained why the *d*_100_(exp.) was lower than the *d*_100_(cal.) for the pimelate ions. For azelate and sebacate ions with ∆*d*_100_/*L* values of −14.8 and −21.2%, respectively, the *d*_100_(exp.) was also lower than the *d*_100_(cal.). However, in this case, this observation implied that the two dicarboxylate ions had slightly bent structures owing to their long and flexible hydrocarbon chains. Advanced calculations, such as those on the dicarboxylate ions in the OCP crystal, would reveal the detailed bent structures of the two carboxylate ions. For the malonate ion, the ∆*d*_100_/*L* value was −19.9%. Since it was unreasonable to believe that this small molecule was compressed by ~20% and packed between the OCP interlayers, it was concluded that our initial assumption of the dicarboxylate ions being parallel to the *a*-axis was not valid for malonic acid. In other words, this result suggests the possibility that previously unknown carboxylic acid arrangements may exist in the OCP interlayers.

The compositions of OCP with incorporated aliphatic dicarboxylate ions are provided in Table S1.

### 3.3. Steric Structures of Succinic Acid Derivatives Bearing Side Chains in OCP

**Figure 4:**
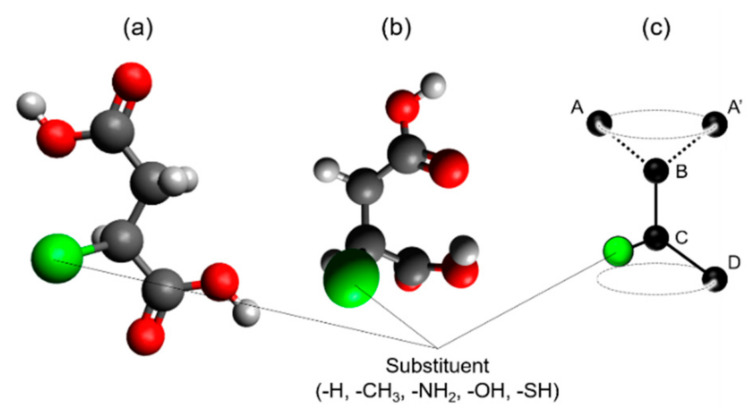
Schematic steric structures of succinic acid derivatives: (**a**) Z-type and (**b**) C-type. (**c**) Carbon atom arrangements of A-B-C-D and A’-B-C-D indicate Z-type and C-type succinic acid derivatives, respectively. The carbon atoms A, A’, and D belong to the carboxyl group.

Additionally, the characteristics of the OCP crystals incorporated with ions of succinic acid derivatives bearing side chains were analysed. In this case, the steric structures of these dicarboxylate ions had to be considered to discuss the relationship between their molecular sizes and the *d*_100_. *L* values were calculated for the dicarboxylic acids having both a linear structure and specific bent structure, which were designated as Z- and C-types, depending on their conformation (Figure 4a,b). Both of the terminal carboxy groups of the succinic acid derivatives were found to rotate around the bond between carbon B and carbon C (Figure 4c). Thus, if the Z- and C-type structures are completely planar, they are considered as the steric structures that provide the maximum and the minimum *L* values, respectively. The findings show the Z-type structures to be almost planar, and thus can be considered to have the highest *L* value. Conversely, the C-type structure was not completely planar due to alleviating steric hindrance caused by the approach of the terminal carboxy group. Thus, the calculated *L* value for the C-type structure is not the minimum value predicted from the ideal C-type structure, but it is close to it. 

The results of the calculations of the Z- and C-type structures of succinic acid and its derivatives are summarised in Table 2. For succinic acid, the *L* values were determined to be 3.86 Å (Z-type) and 3.31 Å (C-type), while the respective ∆*d*_100_/*L* values were 3.0 and 19.3%. As the ∆*d*_100_/*L* of the Z-type structure was lower than that of the C-type structure, the incorporated succinate ions were concluded to have the former structure. The Z- and C-type structures of methylsuccinic acid had *L* values of 3.84 and 3.22 Å, respectively, and the respective ∆*d*_100_/*L* values were −19.8 and −5.5%. Considering the low substitution rate of 25% (Table S2), the incorporated methylsuccinate ions, therefore, featured a C-type structure. The Δ*d*_100_/*L* values of Z-type and C-type aspartic acid were −0.9 and 18.9%, respectively, indicating that the incorporated aspartate ions had the former structure. Similarly, the incorporated malate ion also had a C-type structure. Our calculations indicated that except for mercaptosuccinic acid, the ∆*d*_100_/*L* values were low for one structure (either Z-type or C-type) and high for the other. However, ∆*d*_100_/*L* was high for both Z- and C-type mercaptosuccinic acid, which suggests that the incorporated mercaptosuccinate ions either possessed both Z- and C-type structures or an intermediate structure between Z- and C-types. 

The compositions of OCP with incorporated methylsuccinate, aspartate, malate, and mercaptosuccinate ions are provided in Table S2.

## 4. Conclusions

**Figure 5:**
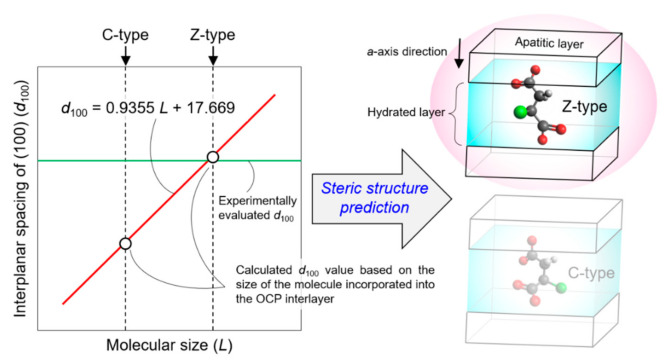
Computationally predicted steric structures of dicarboxylate ions incorporated into the OCP interlayer space.

The steric structures of dicarboxylate ions incorporated into OCP were determined based on the analysis of the relationship between the dicarboxylic acid chain length (*L*) and the (100) interplanar spacing in dicarboxylate-incorporated OCP (*d*_100_), using the computational approach proposed in our previous work (Figure 5). The incorporated succinate, glutarate, adipate, pimelate, and suberate ions were confirmed to possess a linear structure, whereas the incorporated azelate and sebacate ions had a slightly bent structure. Moreover, the steric structure of the incorporated malonate ion was confirmed to be significantly different from those of the other dicarboxylate ions. The incorporated methylsuccinate, aspartate, and malate ions featured C-, Z-, and C-type steric structures, respectively, while the incorporated mercaptosuccinate ions exhibited either Z-type, C-type, or an intermediate structure between the two types. Even though the steric structures of the ions of succinic acid derivatives incorporated into OCP crystals have been revealed, their dependency on the nature of the side chain remains to be explored. Although the steric structures of the incorporated dicarboxylate ions were discussed based on the optimised structures of the isolated dicarboxylic acid molecules, more advanced calculations on incorporated dicarboxylate ions in OCP interlayers are necessary to gain further insights into the crystal structure of OCP with incorporated dicarboxylate ions. The computational approach used in this study to elucidate the understanding of the steric structures of the incorporated dicarboxylate ions, therefore, holds significant potential to facilitate the molecular-level design of novel OCP-based functional materials.

## Figures and Tables

**Table 1:** Values of *d*_100_(exp.), *L*, *d*_100_(cal.), and ∆*d*_100_/*L* for the ions of aliphatic dicarboxylic acids incorporated in the OCP crystal.

Dicarboxylic Acids	Experimental Value	Calculated Value		∆*d*_100_/*L* (%)
	*d*_100_(exp.) (Å)	*L* (Å)	*d*_100_(cal.) (Å)	
Malonic acid	19.6 [24]	2.62	20.1	−19.9
Succinic acid	21.4 [24]	3.86	21.3	+3.0
Glutaric acid	22.3 [24]	5.07	22.4	−2.3
Adipic acid	23.6 [24]	6.40	23.7	−0.9
Pimelic acid	24.4 [24]	7.64	24.8	−5.4
Suberic acid	26.1 [24]	8.95	26.0	+0.6
Azelaic acid	25.7 [24]	10.19	27.2	−14.8
Sebacic acid	26.0 [24]	11.51	28.4	−21.2

**Table 2:** Values of *d*_100_(exp.), *L*, *d*_100_(cal.), and ∆*d*_100_/*L* for the ions of succinic acid and its derivatives incorporated in the OCP crystal.

Dicarboxylic Acids	Experimental Value	Calculated Value			∆*d*_100_/*L* (%)
	*d*_100_(exp.) (Å)	Type of steric structure	*L* (Å)	*d*_100_(cal.) (Å)	
Succinic acid	21.4 [14]	Z C	3.86 3.31	21.3 20.8	+3.0 +19.3
Methylsuccinic acid	20.5 [15]	Z C	3.84 3.22	21.3 20.7	−19.8 -5.5
Aspartic acid	21.3 [15]	Z C	3.91 3.09	21.3 20.6	−0.9 +18.9
Malic acid	20.8 [15]	Z C	3.91 3.28	21.3 20.7	−13.5 +1.8
Mercaptosuccinic acid	21.0 [15]	Z C	3.86 3.08	21.3 20.6	−7.3 +14.5

## Data Availability

The data that support the findings of this study are available from the corresponding author upon reasonable request.

## Supplementary Materials

The following are available online at https://www.mdpi.com/article/10.3390/ma14112703/s1: Table S1: Experimentally determined spacing between the (100) planes in OCP crystals (*d*_100_(exp.)), Ca/P molar ratio, and extent of HPO_4_^2−^ substitution by aliphatic dicarboxylate ions. Table S2: Experimentally determined spacing between the (100) planes in OCP crystals (*d*_100_(exp.)), Ca/P molar ratio, and extent of HPO_4_^2−^ substitution by succinic acid derivatives.

## References

[1] Mathew M., Brown W.E., Schroeder L.W., Dickens B. (1988). Crystal structure of octacalcium bis(hydrogenphosphate) tetrakis(phosphate)pentahydrate Ca_8_(HPO_4_)_2_(PO_4_)_4_·5H_2_O. J. Crystallogr. Spectrosc. Res..

[2] Monma H., Goto M. (1983). Succinate-complexed octacalcium phosphate. Bull. Chem. Soc. Jpn..

[3] Yamada I., Tagaya M. (2019). Immobilization of 2,2′-bipyridine-5,5′-dicarboxylic acid in layered octacalcium phosphate. Colloid Interface Sci. Commun..

[4] Sugiura Y., Makita Y. (2019). Ammonium inhibition of the intercalation of dicarboxylic acid molecules into octacalcium phosphate layer by substitution. J. Solid State Chem..

[5] Yokoi T., Goto T., Kitaoka S. (2018). Transformation of dicalcium phosphate dihydrate into octacalcium phosphate with incorporated dicarboxylate ions. J. Ceram. Soc. Jpn..

[6] Li Y., Reid D.G., Duer M.J., Chan J.C.C. (2018). Solid state NMR-An indispensable tool in organic-inorganic biocomposite characterization; refining the structure of octacalcium phosphate composites with the linear metabolic di-acids succinate and adipate. Solid State Nucl. Magn. Reson..

[7] Yokoi T., Machida S., Sugahara Y., Hashimoto M., Kitaoka S. (2017). Enantioselective incorporation of dicarboxylate guests by octacalcium phosphate. Chem. Commun..

[8] Yokoi T., Kamitakahara M., Ohtsuki C. (2015). Continuous expansion of the interplanar spacing of octacalcium phosphate by incorporation of dicarboxylate ions with a side chain. Dalton Trans..

[9] Davies E., Müller K.H., Wong W.C., Pickard C.J., Reid D.G., Skepper J.N. (2014). Citrate bridges between mineral platelets in bone. Proc. Natl. Acad. Sci. USA.

[10] Yokoi T., Kato H., Kim I.Y., Kikuta K., Kamitakahara M., Kawashita M., Ohtsuki C. (2012). Formation of octacalcium phosphates with co-incorporated succinate and suberate ions. Dalton Trans..

[11] Ishihara S., Matsumoto T., Onoki T., Sohmura T., Nakahira A. (2009). New concept bioceramics composed of octacalcium phosphate (OCP) and dicarboxylic acid-intercalated OCP via hydrothermal hot-pressing. Mater. Sci. Eng. C.

[12] Aoki S., Nakahira A., Nakayama H., Sakamoto K., Yamaguchi S., Suganuma K. (2004). Synthesis and aldehyde absorption properties of aspartate-octacalcium phosphate inclusion compound. J. Phys. Chem. Solids.

[13] Tuncer M., Bakan F., Gocmez H., Erdem E. (2019). Capacitive behaviour of nanocrystalline octacalcium phosphate (OCP) (Ca_8_H_2_(PO_4_)_6_·5H_2_O) as an electrode material for supercapacitors: Biosupercaps. Nanoscale.

[14] Monma H. (1984). The incorporation of dicarboxylates into octacalcium bis(hydrogenphosphate) tetrakis(phosphate) pentahydrate. Bull. Chem. Soc. Jpn..

[15] Aoki S., Sakamoto K., Yamaguchi S., Nakahira A. (2000). Syntheses of octacalcium phosphate containing dicarboxylic acids and effects of the side groups on the crystal growth of octacalcium phosphate. J. Ceram. Soc. Jpn..

[16] Yokoi T., Goto T., Hara M., Sekino T., Seki T., Kamitakahara M., Ohtsuki C., Kitaoka S., Takahashi S., Kawashita M. (2021). Incorporation of tetracarboxylate ions into octacalcium phosphate for the development of next-generation biofriendly materials. Commun. Chem..

[17] Granovsky A.A. (2016). FIREFLY (Version 8.2.0).

[18] Stephens P.J., Devlin F.J., Chabalowski C.F., Frisch M.J. (1994). Ab initio calculation of vibrational absorption and circular dichroism spectra using density functional force fields. J. Phys. Chem..

[19] Becke A.D. (1993). Density-functional thermochemistry. III. The role of exact exchange. J. Chem. Phys..

[20] Lee C., Yang W., Parr R.G. (1988). Development of the Colle-Salvetti correlation-energy formula into a functional of the electron density. Phys. Rev. B.

[21] Vosko S.H., Wilk L., Nusair M. (1980). Accurate spin-dependent electron liquid correlation energies for local spin density calculations: A critical analysis. Can. J. Phys..

[22] Hanwell M.D. (2016). AVOGADRO (Version 1.2.0).

[B23-materials-14-02703] 23.Powder Diffraction file #01-074-1301.

[24] Monma H. (1992). Apatitic intercalation compounds containing dicarboxylates. Gypsum Lime.

