# Supplementary material for: Understanding the Steric Structures of Dicarboxylate Ions Incorporated in Octacalcium Phosphate Crystals
Source: Materials (Basel). 2021 May 21;14(11):2703. doi: 10.3390/ma14112703 (PMC8196614; doi:10.3390/ma14112703)
Supplement: Supplementary file 1 [file materials-14-02703-s001.zip › materials-1205585-supplementary.pdf]

materials

Supplementary Information

Understanding the Steric Structures of Dicarboxylate Ions Incorporated in Octacalcium Phosphate Crystals

Taishi Yokoi * and Masakazu Kawashita

Institute of Biomaterials and Bioengineering, Tokyo Medical and Dental University, 2-3-10 Kanda-Surugadai, Chiyoda-ku, Tokyo 101-0062, Japan; kawashita.bcr@tmd.ac.jp
* Correspondence: yokoi.taishi.bcr@tmd.ac.jp; Tel.: +81-3-5280-8014

Citation: Yokoi, T.; Kawashita, M. Understanding the Steric Structures of Dicarboxylate Ions Incorporated in Octacalcium Phosphate Crystals. *Materials* **2021**, *14*, 2703. https://doi.org/10.3390/ma14112703
Academic Editor: Aivaras Kareiva

Received: 16 April 2021
Accepted: 18 May 2021
Published: 21 May 2021

Publisher's Note: MDPI stays neutral with regard to jurisdictional claims in published maps and institutional affiliations.

Copyright: © 2021 by the authors. Licensee MDPI, Basel, Switzerland. This article is an open access article distributed under the terms and conditions of the Creative Commons Attribution (CC BY) license (http://creativecommons.org/licenses/by/4.0/).

Table S1. Experimentally determined spacing between the (100) planes in OCP crystals (d_{100}(exp.)) [1], Ca/P molar ratio [1], and extent of HPO_4^{2-} substitution by aliphatic dicarboxylate ions.

Dicarboxylic Acid	d_{100}(exp.) (Å)	Ca/P Molar Ratio	Extent of HPO_4^{2-} Substitution (%)
Malonic acid	19.6	1.47	56
Succinic acid	21.4	1.55	84
Glutaric acid	22.3	1.45	48
Adipic acid	23.6	1.56	88
Pimelic acid	24.4	1.41	33
Suberic acid	26.1	1.55	84
Azelaic acid	25.7	1.45	48
Sebacic acid	26.0	1.53	77

The general composition of OCP with incorporated dicarboxylate ions can be represented as $Ca_8(HPO_4)_{2-x}(OOC(CH_2)_nCOO)_x(PO_4)_4{\cdot}mH_2O$ ($0 \le x \le 1$). The extent of HPO_4^{2-} substitution by dicarboxylate ions (%) equals 100% × x.

Table 2. Experimentally determined spacing between the (100) planes in OCP crystals (d_{100}(exp.)) [2], Ca/P molar ratio [2], and extent of HPO_4^{2-} substitution by succinic acid derivatives.

Dicarboxylic Acids	d_{100}(exp.) (Å)	Ca/P Molar Ratio	Extent of HPO_4^{2-} Substitution (%)
Methylsuccinic acid	20.5	1.39	25
Aspartic acid	21.3	1.43	41
Malic acid	20.8	1.48	60
Mercaptosuccinic acid	21.0	1.44	45

The general composition of OCP with incorporated dicarboxylate ions can be represented as $Ca_8(HPO_4)_{2-x}(OOC(CH_2)_nCOO)_x(PO_4)_4{\cdot}mH_2O$ ($0 \le x \le 1$). The extent of HPO_4^{2-} substitution by dicarboxylate ions (%) equals 100% × x.

References

1. Monma, H. Apatitic intercalation compounds containing dicarboxylates, *Gypsum Lime* **1992**, *237*, 108–114.
2. Aoki, S.; Sakamoto, K.; Yamaguchi, S.; Nakahira, A. Syntheses of octacalcium phosphate containing dicarboxylic acids and effects of the side groups on the crystal growth of octacalcium phosphate, *J. Ceram. Soc. Jpn.* **2000**, *108*, 909–914.
